# Neck pain and associated factors in a sample of high school students in the city of Bauru, São Paulo, Brazil: cross-sectional study

**DOI:** 10.1590/1516-3180.2020.0168.R1.30102020

**Published:** 2021-01-11

**Authors:** Alberto de Vitta, Thiago Paulo Frascareli Bento, Priscila de Oliveira Perrucini, Lilian Assunção Felippe, Regina Célia Poli-Frederico, Sergio Marques Borghi

**Affiliations:** I PT, PhD. Physiotherapist and Assistant Professor, Centro Universitário das Faculdades Integradas de Ourinhos, Ourinhos (SP), Brazil.; II PT, MSc. Physiotherapist, Universidade do Sagrado Coração (UNISAGRADO), Bauru (SP), Brazil.; III PT, PhD. Physiotherapist and Assistant Professor, Universidade Anhanguera (UNIDERP), Campo Grande (MS), Brazil.; IV PT, PhD. Physiotherapist and Assistant Professor, Universidade Anhanguera (UNIDERP), Campo Grande (MS), Brazil.; V PhD. Biologist and Assistant Professor, Universidade UNOPAR, Londrina (PR), Brazil.; VI PT, PhD. Physiotherapist and Assistant Professor, Universidade UNOPAR, Londrina (PR), Brazil.

**Keywords:** Adolescent, Neck pain, Epidemiology, Cross-sectional studies., Student, Students at risk, Electronic devices, Mental health problems, Associated factors.

## Abstract

**BACKGROUND:**

Neck pain is a major public health problem.

**OBJECTIVE:**

The aim of the present study was to determine the prevalence of neck pain among high school students and to analyze associations with sociodemographic variables, use of electronic devices, habitual physical activity practices and mental health problems.

**DESIGN AND SETTING:**

Cross-sectional epidemiological study on a sample of high school students in the city of Bauru, São Paulo, Brazil.

**METHOD:**

Participants were selected through cluster sampling in two stages and data were collected via face-to-face interviews. Data collection comprised the following steps: 1. sociodemographic characteristics; 2. use of electronic devices; 3. habitual physical activity levels; 3. mental health; and 4. neck pain.

**RESULTS:**

A total of 1,628 participants were interviewed. The prevalence of neck pain was 49.1% (95% confidence interval, CI 46.7 to 51.5), with 40.4% (95% CI 37.0 to 43.7) in men and 57.5% (95% CI 54.2 to 60.9) in women. The variables associated with in neck pain were: female (prevalence ratio, PR = 2.04), use of cell phone in standing posture (PR = 1.47), use of tablet in sitting posture (PR = 1.72), length of computer use greater than 3 hours/day (PR = 1.54), length of cell phone use greater than 3 hours/day (PR = 1.54), length of tablet use greater than 3 hours/ day (PR = 1.34) and mental health problems (PR = 1.56).

**CONCLUSION:**

There is high prevalence of neck pain among students and striking associations with female sex, use of electronic devices and mental health problems.

## INTRODUCTION

Nowadays, in various activities of daily life and at school and at work, individuals of all ages often use electronic equipment (televisions, computers, video games, mobile phones and tablets).^1^^,^^2^ In a review study, it was shown that 79% of the population between 18 and 44 years old used their cell phones almost all the time.^2^^,^^3^ It has been shown in the literature that systematic use of this equipment has a consequent negative impact on people’s health, such as sleep pattern changes, tiredness, anxiety, depression, overweight, decreased levels of physical activity, headaches, stress and pain in the shoulders, hands, low back and neck.^3^^,^^4^

Neck pain is considered by the World Health Organization (WHO) to be the fourth largest health problem with regard to generation of disability in the general population. It is the eighth largest cause of disability among young people between 15 and 19 years old.^5^^,^^6^^,^^7^

The one-year prevalence of neck pain ranges from 4.8% to 79.5%. These rates may be related to sociodemographic factors,^2^^,^^8^^,^^9^^,^^10^^,^^11^ conditions associated with school (type and weight of the school bag and how it is transported; and the school furniture design),^8^^,^^12^ use of electronic devices (such as televisions, computers, tablets and cell phones)^8^^,^^10^^,^^11^^,^^13^^,^^14^ and mental health problems.^2^^,^^5^^,^^10^^,^^15^

Studies on neck pain and factors associated with this are important because the effects of neck pain may negatively interfere with leisure, sports and school activities.^16^^,^^17^^,^^18^^,^^19^ In addition, adolescent neck pain has been correlated with chronic pain in adulthood and often follows a pattern of recurrent exacerbations and remissions. Adolescent neck pain is thus an important predictor of these problems in later life.^5^^,^^17^

It is important to note that, regarding electronic equipment, especially the use of tablets and cell phones, there is no Brazilian data on this relationship. Therefore, new knowledge on this relationship will contribute to other epidemiological investigations, meta-analyses and systematic reviews.

## OBJECTIVE

The objective of this study was to determine the prevalence of neck pain among high school students and to analyze the associations with sociodemographic variables, use of electronic devices, habitual physical activity practices and mental health problems.

## METHODS

### Ethics

This investigation was approved by the local ethics committee (protocol number 1,972,579; date: March 20, 2017).

### Study design

Through a cross-sectional study, data on 1,628 students from high schools in Bauru, São Paulo, Brazil, were analyzed.

### Participants

This study was based on data collected for the project “*Back pain and associated factors among high school students: a longitudinal study*”, financed by the Research Support Foundation of the State of São Paulo (Fundação de Amparo à Pesquisa do Estado de São Paulo, FAPESP), under procedural number 2016/182837. The subjects comprised adolescents aged 14 to 18 years of both sexes who were attending the first and second years of high school in the mornings in the urban area of Bauru, SP, Brazil.

The sample (n = 1366) was obtained by means of conglomerate sampling in two stages. The primary sampling units were the schools and the secondary sampling units were the classes within the first two years of high school education in the schools that had previously been selected. Thus, the sample of school children was formed by all the students in the classes that formed the secondary sampling units that were selected within the sample of schools that formed the primary sampling units.^20^ The criteria adopted for exclusion of some students from the schools that had been randomly selected for the study were the following: below the age of 14 years; above the age of 18 years; non-submission of informed consent form signed by parents or guardians; and refusal to participate.^20^

Taking into account both the inclusion and the exclusion criteria, the questionnaire of this study was answered by 1628 students between March and June 2017.

### Instruments

Age, gender, skin color, income and marital status were evaluated through self-reported questions: age was divided into three age groups; marital status was categorized as single, married or widowed/separated; skin color was categorized as white, black or brown; and income was grouped into five bands (up to one minimum monthly wage; two to five minimum wages; six to ten minimum wages; 11 to 20 minimum wages; more than 20 minimum wages).^20^^,^^21^

The questions that participants were asked regarding their use of electronic devices (television, computer, tablet or cell phone) were the following:

1. Do you watch television? (a. yes. b. no);
2. How many times a week do you watch television? (a. once or twice. b. three or four times. c. five times. d. more than five times);
3. How many hours a day do you watch television? (a. less than one hour. b. two hours. c. three hours. d. four hours. e. five hours. f. more than five hours a day);
4. Do you use a computer? (a. yes. b. no);
5. What type of computer do you use? (a. desktop. b. laptop.);
6. What is the height of your computer screen? (a. eyes above the midpoint of the screen. b. eyes approximately at the midpoint of the screen. c. eyes below the midpoint of the screen);
7. How many times a week do you use your computer? (a. once or twice. b. three or four times. c. five times. d. more than five times);
8. How many hours a day do you use your computer? (a. less than one hour. b. two hours. c. three hours. d. four hours. e. five hours. f. more than five hours a day);
9. What is your eye-to-screen distance while using your computer? (a. < 20 cm. b. 20 cm to 25 cm. c. 25 cm to 30 cm. d. > 30 cm);
10. Do you use a cell phone? (a. yes. b. no);
11. In which posture do you use your cell phone? (a. standing. b. sitting. c. lying down. d. semi-lying down);
12. What is your average daily time spent using your cell phone? (a. less than one hour. b. two to three hours. c. three to four hours. d. more than four hours);
13. What is your eye-to-screen distance while using your cell phone? (a. < 10 cm. b. 10 cm to 15 cm. c. 15 cm to 20 cm. d. > 20 cm);
14. Do you use a tablet? (a. yes. b. no.);
15. In which posture do you use your tablet? (a. standing. b. sitting. c. lying down. d. semi-lying down);
16. What is your average daily time spent using your tablet? (a. less than one hour. b. two to three hours. c. three to four hours. d. more than four hours);
17. What is your eye-to-screen distance while using your tablet? (a. <10 cm. b. 10 cm to 15 cm. c. 15 cm to 20 cm. d. > 20 cm).^2^^,^^11^

The Baecke Questionnaire of Habitual Physical Activity, in its version validated for use in Brazil, was used to verify the level of habitual physical activity practice.^22^ To classify the students, they were subdivided into quartiles according to the individual total score provided by the instrument, which resulted in the following physical activity groups: sedentary (1^st^ quartile); moderately active (2^nd^ and 3^rd^ quartiles); and active (4^th^ quartile).^22^^,^^23^

Mental health was evaluated using the Strengths and Difficulties Questionnaire (SDQ), in the version validated for use in Brazil by Fleitlich.^24^ The questionnaire contains 25 items that are grouped into five scales (hyperactivity, emotional symptoms, behavioral problems, relationship problems and pro-social behavior) containing five items each. Among these 25 items, 10 relate to skills, 14 relate to difficulties and one is considered neutral. Each of the items can be answered as “false”, “more or less true” or “true”. The score for each of the scales is obtained by summing the scores for the five items, thus generating a score that ranges from 0 to 10. The scores for hyperactivity, emotional symptoms, behavioral problems and peer relationship problems are added together to generate a total score for difficulties, ranging from 0 to 40.

According to the author of the scale, total scores greater than or equal to 20 are considered “abnormal” (clinical), i.e. they indicate that there are great difficulties in relation to what is being evaluated, thus requiring specialized intervention. Scores between 16 and 19 indicate limitations, i.e. that the child or adolescent already has some difficulty that, if not properly cared for, may deteriorate and impair their development. Scores less than or equal to 15 are regarded as normal. These cutoff points have been published in the literature and are available on the internet at www.sdqinfo.com.^24^^,^^25^

Presence of neck pain was assessed by means of the Nordic questionnaire, as validated and adapted for use within Brazilian culture,^26^ through the following question: “In the last twelve months, did you feel any pain or discomfort in your cervical spine?” Neck pain was defined as pain, suffering or discomfort in the area between the occipital bone and the third thoracic vertebra, and between the medial margins of the scapulae.^27^^,^^28^ To make it possible for respondents to better specify the neck region where the pain was, an image of the spinal regions was shown in different colors.^26^

### Data collection procedure

After gaining approval from the State Department of Education and obtaining consent from the adolescents’ parents or guardians, baseline data were collected by undergraduate and postgraduate students between March and June 2017.^21^ On this data collection day, the researcher explained the objectives of the study to the students and informed them that their participation would be voluntary in nature and that they had the right to leave the study at any time and the right to confidentiality of their data. Subsequently, the researcher gave guidance regarding filling out the questionnaire and remained available to deal with any doubts that the students might have had. At the time of answering the questionnaire, there was no communication between the students.^20^

For students who were absent at the time of the initial data collection, three extra visits were made to all of the schools, to collect data. Students who continued to be absent after these visits were considered lost. Students who refused to answer were considered to be refusals.^20^

### Data analysis

The Statistical Package for the Social Sciences, version 18.0 (IBM Corp., Armonk, NY, USA) was used to analyze the data. The data were entered by an undergraduate student who did not participate in the study in any other way. Subsequently, 10% of the questionnaires were randomly chosen to test the accuracy of the data typing, and one error was found and corrected. Another 5% were then randomly chosen and no error was found.

Prevalences, confidence intervals, bivariate analyses and Poisson regression analyses between neck pain and all the independent variables were calculated, including determination of the significance levels and the estimated relative risk of the 95% confidence intervals.

**Figure 1.**
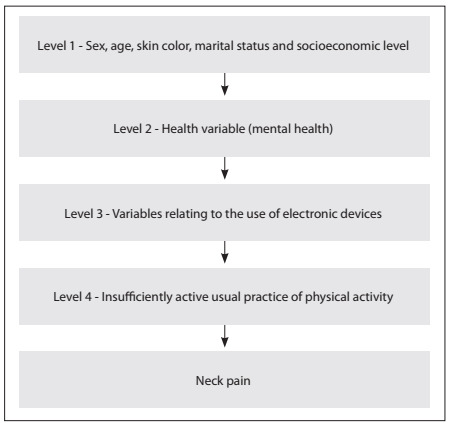
Proposed analysis model for studying neck pain prevalence.

Poisson regression analysis with robust variance was performed in accordance with the theoretical-conceptual hierarchical model. A reference category was established for all variables, which was taken to be the category with the lowest risk. The variables were organized in four levels according to the temporal and causal relationships of neck pain. The adjustment of the first level was performed using all the variables that belonged to this level. The second level was adjusted using variables from the previous level that presented P-values < 0.10, and using those that belonged to the second level. The third level was adjusted using variables from the first and second levels with P-values < 0.10, and using those that belonged to the third level. The fourth level was controlled for the three previous levels (Figure 1). For variables that would remain in the regression model, a regressive selection process was used, such that all the variables with P-values < 0.05 were left in the final model.^29^^,^^30^

## RESULTS

A total of 1628 students were analyzed, after deduction of 2.05% of refusals from the final percentage. Regarding the sociodemographic characteristics of the sample, 51.5% of the males and 53.7% of the females were in the first year of high school; 87% of the males and 82.5% of the females were in the age group of 15 to 18 years; 47.4% of the males and 51.9% of the females were white; and 85.9% of the males and 97.2% of the females were single. Regarding the level of physical activity, most of the males (46.5%) and females (50.7%) were classified as sufficiently active, while 16.4% of the males and 35.7% of the females were insufficiently active. Regarding the mental health variable, 68.7% of the males and 42.3% of the females were considered normal, while 11.3% of the males and 30.0% of the females were classified as “clinically abnormal”.

Table 1 shows the data on the use of electronic devices by these adolescents.

**Table 1.** Distribution of absolute and relative frequencies of use of electronic devices among high school adolescents according to sex

Factors	Sex			
	Male (n = 798)		Female (n = 830)	
	n	%	n	%
Do you watch television?				
No	123	15.4	67	8.1
Yes	675	84.6	763	91.9
How many times do you watch television per week				
Up to 2 times	168	24.7	205	21.1
3 times or more	507	67.2	558	63.5
Number of hours of television/day				
Up to 2 hours	383	48.0	376	45.3
3 hours or more	292	36.6	387	46.6
Do you use a computer?				
No	105	13.2	215	25.9
Yes	693	86.8	615	74.1
What type(s) of computer?				
Desktop	344	43.1	224	27.0
Laptop	263	33.0	339	40.8
Desktop and laptop	86	10.8	52	6.3
Height of the computer screen				
Eye level above the screen midpoint	153	19.2	114	13.7
Eye level at the screen midpoint	473	59.3	435	52.4
Eye level below the screen midpoint	67	8.4	66	8.0
How many times do you use a computer/week?				
Up to 2 times	184	23.1	295	35.5
3 times or more	509	63.8	320	38.6
How many hours of computer use/day?				
Up to 2 hours	250	31.3	341	41.1
3 hours or more	443	55.5	274	33.0
Do you use a cell phone?				
No	33	4.1	9	1.1
Yes	765	95.9	821	98.9
What is your posture when using your cell phone?				
Standing	276	34.6	282	34.0
Sitting	403	50.5	441	53.1
Lying down	436	54.6	491	59.2
Semi-lying down	215	26.9	344	41.4
Daily length of cell phone use				
Up to 2 hours	220	27.6	125	15.1
3 hours or more	545	68.3	696	83.9
Do you use a tablet?				
No	656	82.2	649	78.2
Yes	142	17.8	181	21.8
What is your posture when using your tablet?				
Standing	25	3.0	47	5.6
Sitting	83	10.4	102	12.3
Lying down	68	8.5	82	9.9
Semi-lying down	26	3.3	56	6.7
Daily length of tablet use				
Up to 2 hours	94	11.8	149	18.0
3 hours or more	48	6.0	32	3.9

The overall prevalence of neck pain was 49.1% (95% CI 46.7 to 51.5), while it was 40.4% among the males (95% CI 37.0 to 43.7) and 59.6% among the females (95% CI 54.2 to 60.9).

The bivariate analysis **(**Table 2**)** showed that neck pain was associated with female sex and mental health problems.

**Table 2.** Bivariate analysis on neck pain in relation to sociodemographic characteristics, physical activity level and mental health problems among adolescents

Factors	Neck pain		
	n	%	PR (95% CI)^*^
Gender			
Male	322	40.2	1.00
Female	478	59.8	1.43 (1.29-1.58)
Age range			
14 years	133	16.6	1.00
15 to 18 years	667	83.4	0.91 (0.80-1.03)
Marital status#			
Married	28	3.5	1.00
Single	772	96.5	0.98 (0.75-1.28)
Race			
White	407	50.9	1.00
Black	57	7.1	0.79 (0.64-0.98)
Brown/mixed	287	35.9	0.99 (0.89-1.10)
East Asian	29	3.6	0.98 (0.75-1.28)
Indigenous	20	2.5	1.05 (0.77-1.34)
Level of physical activity			
Very active	185	23.1	1.00
Sufficiently active	387	48.4	1.07 (0.94-1.22)
Insufficiently active	217	27.1	1.13 (0.98-1.29)
Mental health problems			
Normal	362	45.3	1.00
Borderline	214	26.8	1.46 (1.30-1.64)
Clinical	213	26.6	1.56 (1.39-1.75)

^*^Adjusted according to age and/or sex; ^#^The legal age for marriage in Brazil is 16 years (Law No. 13,811/19 of the Brazilian Civil Code).

PR = prevalence ratio; CI = confidence interval.

Neck pain was significantly associated with use of a cell phone in a standing posture and when semi-lying down, with daily use of a cell phone totaling more than three hours and with use of a tablet and its use in a sitting posture **(**Table 3**).**

**Table 3.** Bivariate analysis on neck pain in relation to use of electronic devices among adolescents

Factors	Neck pain		
	n	%	PR (95% CI)^*^
Do you watch television?			
No	99	12.4	1.00
Yes	701	87.6	0.94 (0.81-1.08)
How many times do you watch television per week?			
Up to 2 times	177	22.1	1.00
3 times or more	524	65.5	1.04 (0.92-1.17)
Number of hours of television/day			
Up to 2 hours	361	45.1	1.00
3 hours or more	340	42.5	1.05 (0.95-1.17)
Do you use a computer or videogame?			
No	143	17.9	1.00
Yes	657	82.1	1.12 (0.98-1.28)
What type(s) of computer?			
Desktop	297	37.1	1.00
Laptop	323	40.4	1.00 (0.89-1.11)
Desktop and laptop	37	4.6	1.02 (0.81-1.30)
Height of the computer screen			
Eye level above the screen midpoint	164	20.5	1,00
Eye level at the screen midpoint	427	53.4	1,00 (0,88 - 1,14)
Eye level below the screen midpoint	66	8.3	0,99 (0,81 - 1,21)
How many times do you use a computer/week?			
Up to 2 times	247	30.9	1.00
3 times or more	410	51.3	0.96 (0.86-1.08)
How many hours of computer use/day?			
Up to 2 hours	287	35.9	1.00
3 hours or more	370	46.3	1.07 (0.96-1.19)
Do you use a cell phone?			
No	17	2.1	1.00
Yes	783	97.9	1.22 (0.84-1.77)
What is your posture when using your cell phone?			
Standing			
No			1.00
Yes	316	39.5	1.23 (1.11-1.36)
Sitting			
No			1.00
Yes	435	54.4	1.10 (0.99-1.22)
Lying down			
No			1.00
Yes	459	57.4	1.01 (0.91-1.11)
Semi-lying down			
No			1.00
Yes	303	37.9	1.16 (1.05-1.28)
Daily length of cell phone use			
Up to 2 hours	145	18.1	1.00
3 hours or more	638	79.8	1.22 (1.07-1.40)
Do you use a tablet?			
No	613	76.6	1.00
Yes	187	23.4	1.23 (1.10-1.38)
What is your posture when using your tablet?			
Standing			
No			1.00
Yes	48	6.0	1.20 (0.99-1.47)
Sitting			
No			1.00
Yes	119	14.9	1.31 (1.07-1.60)
Lying down			
No			1.00
Yes	90	11.3	1.07 (0.89-1.29)
Semi-lying down			
No			1.00
Yes	52	6.5	1.13 (0.93-1.38)
Daily length of tablet use			
Up to 2 hours	142	17.8	1.00
3 hours or more	45	5.6	0.96 (0.77-1.20)

^*^Adjusted according to age and sex.

PR = prevalence ratio; CI = confidence interval.

In the analysis on multiple factors, after adjustment through logistic regression according to the hierarchical model, cervical pain remained associated with: female gender, length of use of a computer greater than three hours per day, use of a cell phone in a standing posture, use of a cell phone totaling more than three hours per day, use of a tablet totaling more than three hours per day, use of a tablet in a standing posture, use of a tablet in a sitting posture and mental health problems (Table 4).

**Table 4.** Multivariate logistic regression, for associations of variables with neck pain among adolescents

Factors	Neck pain	
	P-value	Adjusted PR (95% CI)
Sex^*^		
Male	0.001	1.00
Female		2.04 (1.66-2.07)
Use of a tablet in a standing up posture^**^		
No	0.01	1.00
Yes		1.54 (1.25-1.90)
Use of a cell phone in a standing up posture^**^		
No	0.001	1.00
Yes		1.47 (1.21-2.50)
Use of a tablet in a sitting posture^**^		
No	0.01	1.00
Yes		1.72 (1.09-2.77)
Daily length of cell phone use^**^		
Up to 2 hours	0.001	
3 hours or more		1.26 (1.08-1.98)
Daily length of computer use^**^		
Up to 2 hours	0.03	1.00
3 hours or more		1.14 (1.01-1.30)
Daily length of tablet use^**^		
Up to 2 hours	0.002	1.00
3 hours or more		1.34 (1.11-1.61)
Mental health problems^***^		
Normal	0.001	1.00
Borderline		1.01 (0.57-1.80)
Clinical		2.32 (1.28-4.19)

^*^Adjusted for demographic and socioeconomic variables; ^**^adjusted for the variables of the first and second stages and for the variables relating to use of electronic equipment; ^***^adjusted for the first-stage variables and mental problems.

CI = confidence interval; PR = prevalence ratio.

## DISCUSSION

It was found in this study that 41.9% of the students reported having neck pain. This finding was similar to what had been reported in Shanghai, China (40.8%),^2^ Taiwan (46.4%)^11^ and Thailand (44.7%),^12^ while it was lower than in Las Vegas, United States (67.9%)^8^ and Korea (81.6%)^10^ and higher than in Saudi Arabia (23.7%)^13^ and Australia (27.5%).^15^ Sociocultural, demographic, economic and professional differences may have influenced the prevalence rates in these various locations.^20^

The outcome remained associated with female gender, length of use of a computer greater than three hours per day, use of a cell phone in a standing posture, use of a cell phone totaling more than three hours per day, use of a tablet totaling more than three hours per day, use of a tablet in a standing posture, use of a tablet in a sitting posture and mental health problems.

Neck pain was associated with female gender, thus corroborating the findings from other studies on adolescents.^2^^,^^12^^,^^14^ This gender-based difference may be related to a lower pain threshold among women, hereditary factors and higher mental stress among women, besides the fact that they present less strength and smaller body size than men.^2^^,^^8^^,^^10^^,^^31^

Use of computers, cell phones and tablets for more than three hours a day was associated with the outcome of neck pain, and this lined up well with the findings from other investigations.^2^^,^^11^^,^^13^^,^^32^ As mentioned above, the mechanism for pain development may be related to an association of inappropriate postures, static work and repetitive movements in manual activities. In addition, gender, type of activity, levels of physical and mental health and family relationships can contribute to higher numbers of hours of use of electronic equipment.^32^^,^^33^

Neck pain was associated with use of cell phones and tablets in a standing posture. Using electronic equipment in a standing position causes individuals to perform greater cervical flexion when looking at the screen. This increases the muscle activity of the cervical extensors, which is a significant risk factor for cervical pain.^34^

The sitting posture when using a cell phone was a factor associated with neck pain, and this corroborated data from other studies.^4^ It was previously shown that for the head to be stabilized and maintained in an upright position, there is a need for greater activity of the cervical and thoracic extensor muscles.^8^ This prolonged isometric contraction of the cervical extensors promotes increased muscle tension and stress, thus causing pain. In addition, postures with greater cervical flexion, such as using a cell phone resting on one’s lap or on a table, further increase the cervical extensor tension.^8^^,^^9^

Neck pain was associated with a clinical category relating to mental health problems, similar to what was observed in other investigations.^15^^,^^35^^,^^36^ High levels of mental problems are related to increased muscle tension, which possibly affects the nutrition of intervertebral discs, nerve roots and other vertebral tissues. This also leads to adoption of incorrect postures and gives rise to a set of adverse events such as ineffective survival strategies, anxiety, depression, diet, sleep and sedentariness, i.e. a set of factors that contribute to muscle and joint pain.^35^^,^^36^^,^^37^

### Limitations

This study presents some limitations, given that it was based on interviews and, thus, response and memory bias may have occurred. Because of the cross-sectional nature of this study, it was not possible to know the random direction of the pain and mental problem variables. A longitudinal investigation would be required in order to resolve this issue: this was address by our research group through following up these students. Another limitation was that our students came from public schools, which limits the possibility for generalization of these data to students at private schools.

The strengths of this study were that this was one of the first Brazilian investigations to examine the role of factors relating to use of electronic and mental health equipment in the appearance of cervical pain in young people; is use of a validated questionnaire to evaluate the results; and the large number of students interviewed.

## CONCLUSIONS

Collectively, it was concluded that neck pain had high prevalence and striking associations with the following: female gender; length of use of a computer greater than three hours per day; use of a cell phone in a standing posture; use of a cell phone for more than three hours per day; use of a tablet for more than three hours per day; use of a tablet in a standing posture; use of a tablet in a sitting posture; and mental health problems.
